# Astaxanthin Prevents Human Papillomavirus L1 Protein Binding in Human Sperm Membranes

**DOI:** 10.3390/md16110427

**Published:** 2018-11-02

**Authors:** Gabriella Donà, Alessandra Andrisani, Elena Tibaldi, Anna Maria Brunati, Chiara Sabbadin, Decio Armanini, Guido Ambrosini, Eugenio Ragazzi, Luciana Bordin

**Affiliations:** 1 Department of Molecular Medicine-Biological Chemistry, University of Padova, 35131 Padova, Italy; gabriella.dona@unipd.it (G.D.); elena.tibaldi@unipd.it (E.T.); annamaria.brunati@unipd.it (A.M.B.); 2 Department of Women’s and Chilren’s Health, University of Padova, 35131 Padova, Italy; alessandra.andrisani@unipd.it (A.A.); guido.ambrosini@unipd.it (G.A.); 3 Department of Medicine-Endocrinology, University of Padova, 35131 Padova, Italy; ChiaraSabbadin@libero.it (C.S.); decio.armanini@unipd.it (D.A.); 4 Department of Pharmaceutical and Pharmacological Sciences, University of Padova, 35131 Padova, Italy; eugenio.ragazzi@unipd.it

**Keywords:** human papillomavirus 16 (HPV16), astaxanthin (Asta), acrosome reaction, cholera toxin subunit B (CTB), L1 protein

## Abstract

Astaxanthin (Asta), red pigment of the carotenoid family, is known for its anti-oxidant, anti-cancer, anti-diabetic, and anti-inflammatory properties. In this study, we evaluated the effects of Asta on isolated human sperm in the presence of human papillomavirus (HPV) 16 capsid protein, L1. Sperm, purified by gradient separation, were treated with HPV16-L1 in both a dose and time-dependent manner in the absence or presence of 30 min-Asta pre-incubation. Effects of HPV16-L1 alone after Asta pre-incubation were evaluated by rafts (CTB) and Lyn dislocation, Tyr-phosphorylation (Tyr-P) of the head, percentages of acrosome-reacted cells (ARC) and endogenous reactive oxygen species (ROS) generation. Sperm membranes were also analyzed for the HPV16-L1 content. Results show that HPV16-L1 drastically reduced membrane rearrangement with percentage of sperm showing head CTB and Lyn displacement decreasing from 72% to 15.8%, and from 63.1% to 13.9%, respectively. Accordingly, both Tyr-P of the head and ARC decreased from 68.4% to 10.2%, and from 65.7% to 14.6%, respectively. Asta pre-incubation prevented this drop and restored values of the percentage of ARC up to 40.8%. No alteration was found in either the ROS generation curve or sperm motility. In conclusion, Asta is able to preserve sperm by reducing the amount of HPV16-L1 bound onto membranes.

## 1. Introduction

HPV (Human Papilloma Virus) is responsible for the 5.2% (3% in women and 2% in men) of cancers in the world, with prevalence in cervical, ano-genital, head, and neck cancers [1]. Oncogenic strains of HPV DNA were demonstrated in almost all cervical malignancies in women [2], and, on a global scale, also in 5% of men [3], in the form of penile cancer (45%) with the HPV 16 member mostly responsible (60%) followed by the HPV 18 accounting for 13% of the cases [4].

Besides sexual inter-infections between sexual partners leading to the wider virus propagation, it has been recently shown that HPV DNA can cause a detrimental effect on early embryo development and clinical reproductive outcomes, since HPV DNA can be harbored inside the blastocyst stage by spermatozoa carrying HPV virions, which represent the viral DNA included inside the L1–L2 capsid [5,6,7]. 

HPV infection is considered a cause of male infertility or subfertility [8], even if the mechanism of the reduced sperm motility and DNA degradation is still debated [9]. HPV is a non-enveloped double stranded DNA virus with a genome of 8kb pairs encoding two protein types: (i) the “Late proteins” L1 and L2 which are the structural components of viral capsid and are involved in the packaging of the virus; and (ii) the “Early proteins” E1,2,4,5,6,7 which regulate the replication of viral DNA. The early proteins are expressed throughout the life cycle of the virus, whereas the late proteins are expressed only during the initial stages of infection [10]. During the uncoating (process needed for the releasing of the viral genomes into the host nuclei), the protective capsids undergo sequential structural changes. HPV16 L1 binds primarily to heparan sulfate proteoglycans (HSPGs) on the host cell, in particular with glycosaminoglycan (GAG) chains, Upon HSPG binding, capsid goes through conformational changes that are required for a secondary binding event which, in turn, triggers the uptake of the virus through a still unknown receptor [10]. One of these conformational changes leads to the exposition of the L2 protein on the capsid surface followed by a furin convertase dependent cleavage of the L2 N-terminus. Only upon L2 cleavage can the virus capsids be transferred from the membrane to a secondary receptor on the cell surface, and, hence, be endocytosed via receptors [10]. 

L1, the main constituent of the capsid envelope, spontaneously self-assembles into virus-like particles (VLPs), that are an icosahedral structure composed of 72 pentamers of L1 and an unknown number of the L2 minor coat proteins. Due to its ability of self-assembling in VLPs also in the absence of the L2 protein, L1 has been considered for vaccine development against HPV infections, since the L1-based VLPs share the same immunogen properties of native HPV, but lack genome and other proteins [10,11].

Astaxanthin (Asta) is mainly produced by the microalgae *Haematococcus pluvialis* in the presence of stressing conditions including, deficiency of nitrogen, high salinity, and high temperature [12].

For its molecular structure, Asta belongs to the carotenoid family, with an extended nonpolar zone in the middle, which is made up of a series of carbon–carbon double bond termed “conjugated atoms” and two polar regions at either ends. This nonpolar–polar structure allows Asta to fit precisely into the polar–nonpolar-polar area of the cell membrane [13,14] with a suitable capacity for neutralizing free radicals, which is 65 times more powerful than vitamin C, 54 times stronger than β-carotene, and 100 times more effective than α-tocopherol [14,15]. Asta has a wide range of applications in the food, feed, cosmetic, aquaculture, nutraceutical, and pharmaceutical industries. In the last decades, Asta was described to have anti-inflammatory and pain relieving activity, due to its ability of blocking different biochemical factors involved in pain [14]. More specifically, Asta inhibits cyclooxygenase 2 (COX2) enzyme activities, which are related to many diseases, such as osteoarthritis, rheumatoid arthritis, dysmenorrhea, and acute pain [14]. Asta and *H*. *pluvialis* extracts prevents the development of human colon cancer cells by blunting the progression of the cell cycle, ameliorating apoptosis, and suppressing the expression of inflammatory cytokines (e.g., NF-kβ, TNF-a and IL-1β) [15,16,17,18]. In HCT116 and HT29 cells, Asta induced the expression of the negative regulators of the cell cycle [19]. 

Recent evidence has demonstrated that Asta treatment improves human sperm capacitation by inducing the membrane rafts relocation [20,21]. During the process of capacitation sperm undergoes a series of transformations, including reactive oxygen species (ROS) production, membrane micro-domains rafts translocation, Lyn displacement and activation, and sperm head Tyr-phosphorylation (Tyr-P) [20,21,22,23,24]. This transformation is needed to let sperm undergo the acrosome reaction, with the release of lytic enzymes responsible for sperm-oocyte fusion occurring.

The aim of this study was to evaluate the effect of HPV capsid protein L1 on human sperm capacitation, and the effect of Asta in L1 binding to plasma sperm membranes.

## 2. Results and Discussion

### 2.1. L1 Treatment: Effect on Sperm L1 Location

**Figure 1:**
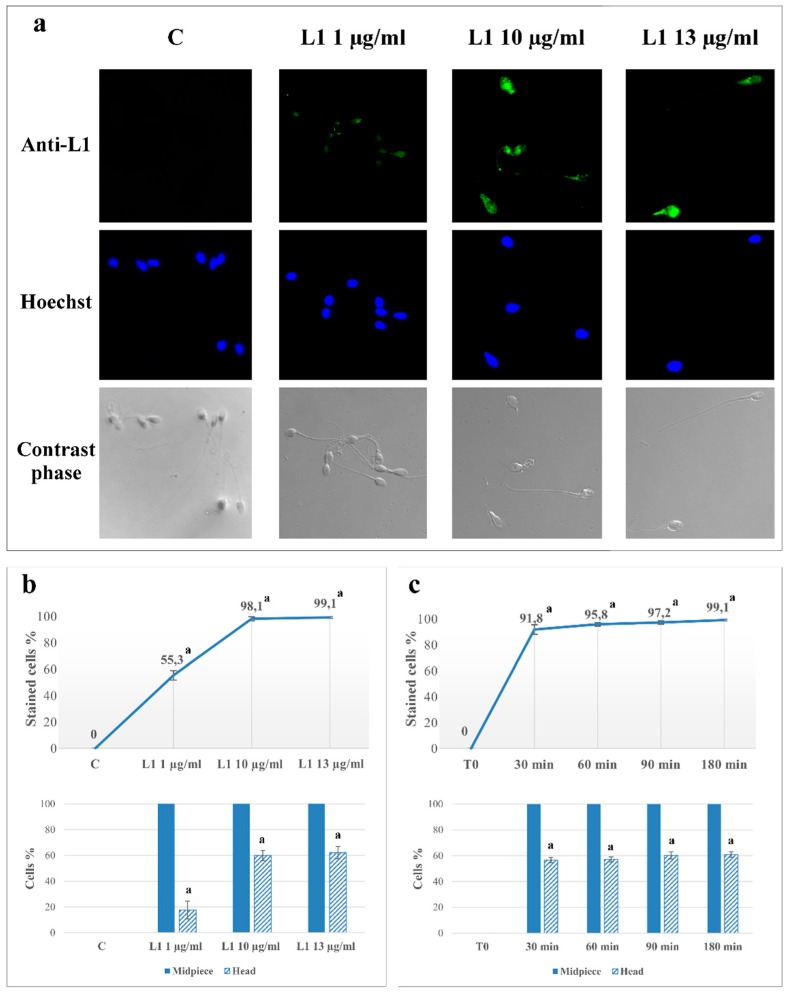
Before (T_0_) or after (T_1_) 180 min of incubation in capacitating conditions in absence (C) or presence of L1 (1, 10 or 13 μg/mL), sperm were analyzed for L1 presence by immunofluorescence cytochemistry as described in Methods. Similarly, time-dependent evaluation was carried out with L1 (10 μg/mL) for 30, 60, 90, 180 min. (**a**) Immunofluorescence analysis was performed with anti-L1 antibody (green) and Hoechst (blue) was used to visualize nuclei. Corresponding phase-contrast images for each condition were shown. (**b**) Dose dependent evaluation of protein L1 localization in C, L1 1 μg/mL, L1 10 μg/mL, and L1 13 μg/mL samples. The graph in the upper side shows the percentage of cell marked by L1 protein, whereas the histogram at the bottom shows L1 distribution between the mid-piece (filled blue) and the head (striped). (**c**) Time dependent evaluation of protein L1 localization in sample L1 10 μg/mL at different incubation times (30, 60, 90, 180 min). The number of sperm stained with anti-L1 antibody in any part of the cells (stained) is expressed as % of the total number of cells analyzed, whereas the number of cells showing L1 in the head or mid-piece is expressed as means ± SD% of marked cells. The figure is representative of 12 separate experiments conducted in triplicate (^a^: *p* < 0.0001 comparing each sample against C values, by using Dunnett’s test, following a significant one-way ANOVA).

Aliquots of sperm, isolated and pooled as described in Methods, were analyzed for L1 protein binding and distribution with immunocytochemistry, immediately (T_0_), or after incubation in capacitating conditions, in the absence (C), or presence of 1 (L1 1), 10 (L1 10) or 13 (L1 13) µg/mL of L1 protein (Figure 1a). The expected absence of anti-L1 fluorescence in T_0_ and C confirmed that samples were free from any HPV pre-existing infection. Conversely, L1-capacitated sperm fluorescence indicated that L1 bound to cells in a concentration-dependent way, with a percentage of marked cells at 1, 10 and 13 μg/mL reaching 55.3% ± 3.6%, 98.1% ± 1.6% and 99.1% ± 0.7% of cells compared to control 0%, respectively (*p* < 0.0001) (Figure 1b). Due to its ability to bind to all sperm, a concentration of 10 μg/mL was chosen as suitable concentration to further investigate the time-dependent effect.

Experiments with L1 10 µg/mL were carried out for 30, 60, 90, 180 min of incubation and sperm fluorescence evaluated. Interestingly, already after 30 min of L1 incubation, 91.8% ± 3.7% of sperm presented L1 related fluorescence (*p* < 0.0001 compared to control), which was progressively more marked by prolonging incubation time up to 3 h.

A qualitative analysis was also performed to evaluate the main site of protein binding (bottom panels of b and c). In the dose-dependent experiments, almost 100% of the marked cells showed fluorescence at the mid-piece, eligible as the principal binding point right after the first 30 min of incubation with 10 μg/mL, HPV L1 (Figure 1c). Interestingly, with 1 μg/mL only 20% of the marked cells showed L1 binding also in the head, whereas the maximal effect was observed with 10 μg/mL (60%). The L1 distribution between mid-piece and head did not change with incubation time (Figure 1, panels b and c).

### 2.2. L1 Treatment: Effect on Sperm Motility

Aliquots were then assessed for motility by computer-assisted sperm analysis (CASA) (Table 1). Samples were incubated in the presence of increasing L1 concentration (1, 10, and 13 µg/mL as representing the concentrations able to infect 100% of cells), after a pre-incubation of 30 min in the presence or absence of 2 μM Asta [20,21]. The increase of values of all parameters (except motility) is evident with all treatments compared to T_0_, meaning that capacitation had occurred leading to hyper-motility. However, when values from treated cells were compared to C (capacitated without Asta and/or L1), there was no significant difference, also for samples pre-incubated alone or with Asta and L1.

These results suggest that capacitated-related hyper-motility was not affected by Asta, by L1, or by both Asta and L1.

### 2.3. Effect of L1 and Asta Alone or in Association on Sperm Capacitation

We previously showed that capacitation of sperm is linked to membrane rearrangement with lipid rafts relocation to the apical part of the head [20]. This rearrangement allows the gathering of the src kinase family member, Lyn, which activates and increases the Tyr-phosphorylation of the sperm head proteins [21]. To evidence the effects of L1 on sperm capacitation parameters, aliquots of sperm treated with L1 (10 μg/mL), after a pre-incubation of 30 min in the presence or absence of Asta, as described above, were analyzed for their shifted rafts (CTB), Lyn location, Tyr-P, and acrosome reaction (ACR).

**Figure 2:**
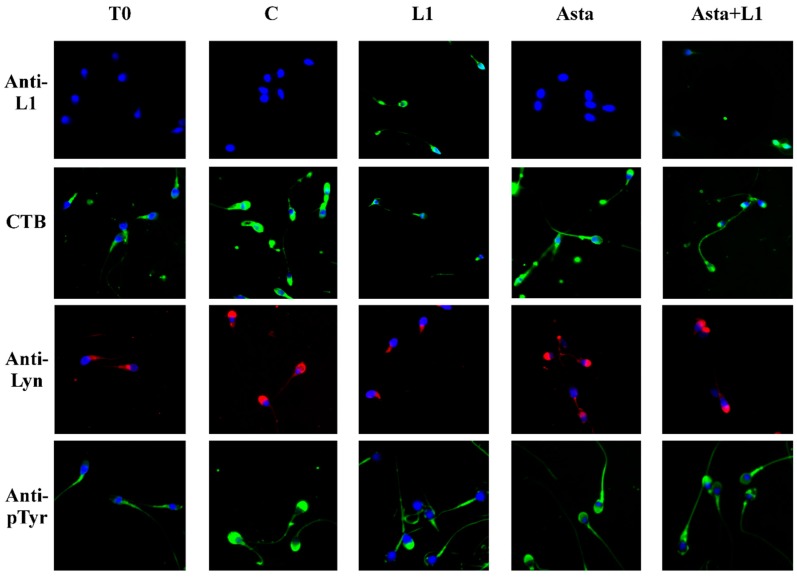
Localization of L1 protein, membrane rafts, Lyn and Tyr-P protein in human sperm during capacitation in absence or presence of L1 and/or Asta. Sperm cells, at T_0_ or incubated in capacitating conditions for 180 min in absence (C) or presence of L1 10 μg/mL, Asta 2 μM or both, were analyzed for L1 localization (green), CTB labelling (green), Lyn (red) and Tyr-P (green) localization by immunofluorescence cytochemistry as described in Methods. Hoechst (blue) was used to visualize nuclei and images were merged. The figure is representative of 12 separate experiments.

L1 incubation affected rafts relocation (Figure 2, CTB), with the sample showing only 20% of sperm with rafts relocated on the head (sample L1 compared to C). The corresponding quantification is in Table 2 (21.3% ± 4.2% compared to 72.0% ± 3.6% for L1 10 µg/mL and C, respectively, *p* < 0.001). 

The lack of translocation causes the consequent decrease of Lyn gathered on the apical part of the head (Figure 2, Anti-Lyn) (Table 2, 16.3% ± 3.5% compared to 63.1% ± 2.5% for L1 10 µg/mL and C, respectively, *p* < 0.001), followed by the net reduction of the percentage of cells showing Tyr-P of the head (Figure 2, Anti-P-Tyr) (Table 2).

In these conditions only a reduced percentage of cells underwent the acrosome reaction compared to the C sample (19.4% ± 2.8% of acrosome-reacted cells (ARC) in the L1 10 µg/mL sample compared to 65.7% ± 4.2% in C sample, *p* < 0.001) (Table 2).

When Asta was added to the capacitating medium, the values of capacitation only slightly improved, as previously shown [21], since sperm were already in optimal conditions and did not need membrane raft translocation (CTB), Lyn gathering, and Tyr-P. Asta also prevented L1 binding to the membranes of sperm (Figure 2 Anti-L1, compare L1 with Asta+L1 panels) with a net reduction of protein of about 52% (98.1% ± 1.6% of sperm infected by L1 compared to 45.5 ± 4.2% in the presence of Asta, *p* < 0.001, Table 2). In the same way, also Lyn relocation and the following Tyr-P of the head were partially restored. Our results show that Asta can recover about 50% of the L1-induced alterations, with cell showing rafts relocation switching from 21.3% ± 4.2% to 50.2% ± 2.7% (*p* < 0.001), Lyn relocation from 16.3% ± 3.5% to 43.2% ± 3.0% (*p* < 0.001) and Tyr-P of the head from 11.3% ± 1.7% to 43.6% ± 3.9% (*p* < 0.001) (Table 2, comparing line L1 10 µg/mL to Asta+L1 10). Consequently, also the L1-induced reduction of the ARC percentage was greatly restored by the presence of Asta (Table 2), shifting from 19.4% ± 2.8% to 40.8% ± 2.6% (*p* < 0.001), which, also if still far from the 65.7% ± 4.2% of the C sample, accounts for twice the value of the L1-treated sample (L1 10 μg/mL: 19.4% ± 2.8%).

### 2.4. Effect of Asta on L1 Binding to Sperm Membranes

Samples were also analyzed for the L1 protein binding to the subcellular fractions. After incubation in the absence or presence of Asta, L1, or both, aliquots from each sample were sonicated, sub-cellular fractions were separated on gradient and further centrifuged, as described in Methods. Membranes, cytosol, heads, and flagella were subjected to Western blotting and revealed with ant-L1 antibodies.

**Figure 3:**
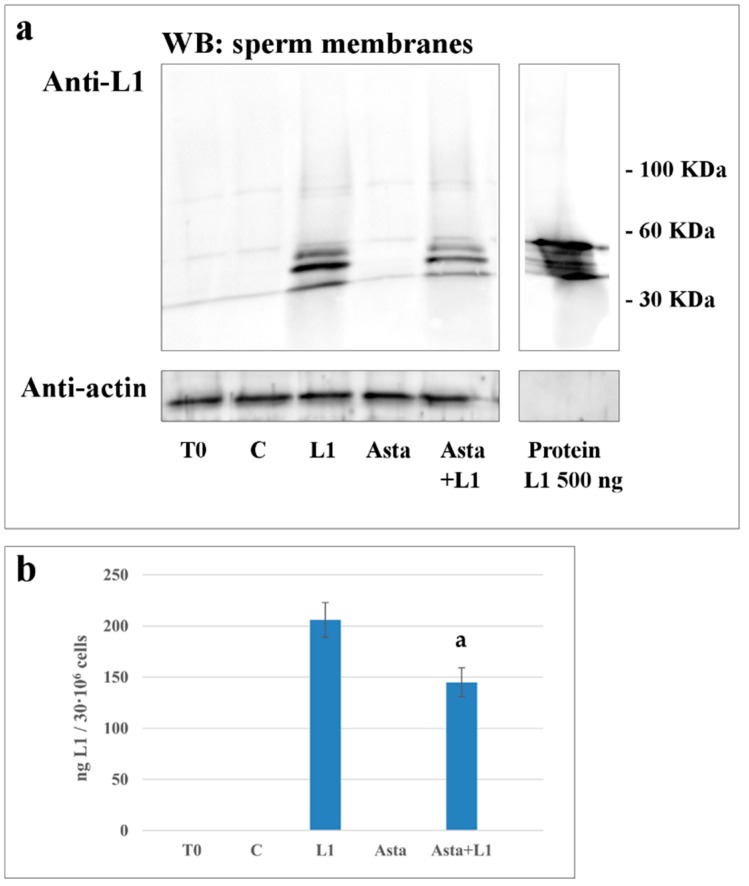
Detection of protein L1 in membrane of human sperm during capacitation in absence or presence of L1 and/or Asta. Western blot analysis (panel **a**) of plasma membrane (obtained as described in Methods) of sperm cells, at T_0_ or incubated in capacitating conditions for 180 min in absence (C) or presence of L1 10 μg/μL, Asta 2 μM or Asta+ L1. Membranes of different samples were analyzed by SDS-PAGE, transferred to nitrocellulose and immuno-revealed with anti-L1 antibody and then with anti-β actin as loading control. Bands were densitometrically analyzed (panel **b**) and the amount of bound L1 was expressed as ng calculated by the ratio between samples and the bands of L1 protein (500 ng). a: *p* < 0.001 comparing Asta+L1 sample to L1. Values are expressed as the mean ± SD. The figure is representative of seven separate experiments conducted in triplicate. When analyzed in other compartments, L1 detection was practically negligible (Figure S1), thus indicating that the main site of L1 binding was on the membrane.

L1 was detected on membranes from L1-treated sperm, (Figure 3, lanes L1 and Asta+L1), but not in T_0_, C and Asta samples did not show any response, as expected. The sample treated with Asta+L1 showed a reduction in the L1 content, with Asta reducing the amount of L1 by about 30%, compared to L1 sample (206 ± 17 vs. 145 ± 14 ng of L1 protein/30 × 10^6^ cells *p* < 0.001). 

When analyzed in other compartments, such as heads and tails, L1 detection was practically negligible (Figure S1), thus indicating that the main site of L1 binding was on the membrane (Figure S2) 

Compared to data in Figure 1 and Table 2, showing that Asta decreased the percentage of marked cells from 98.1% ± 1.6% to 45.5% ± 4.2% (*p* < 0.001) (Table 2), data from the Western blotting of membranes confirmed the net reduction of the amount of L1 in the presence of Asta. The decrease seems not to account for the higher reduction found with immunofluorescence. We must consider that, when evaluated with immunocytochemistry, we account for the number of cells presenting L1-related fluorescence, without considering the amount of the protein effectively linked. By observing the L1 distribution between mid-piece and head, it is reasonable that the binding of L1 starts from the mid-piece and gradually spreads over the whole cell, thus increasing the concentration of L1 for each cell. 

### 2.5. Effect of Asta and L1 on ROS Production

**Figure 4:**
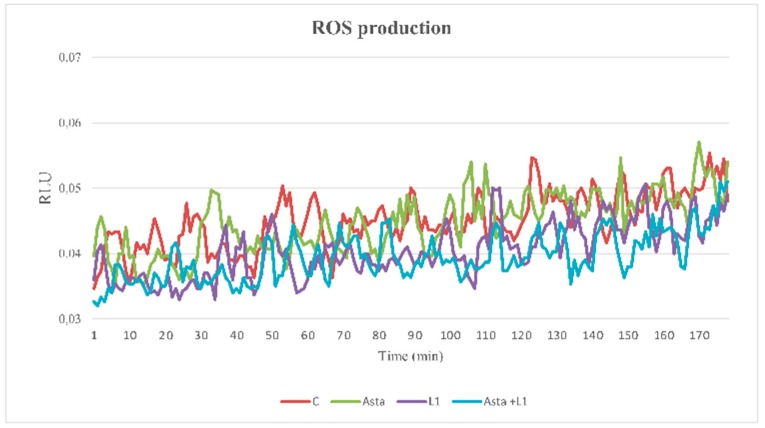
Reactive oxygen species (ROS) generation curves of sperm samples and effects of Asta, L1, or both. Sperm cells from 3 volunteers for each experiment were collected to form a pool with a sufficient number of cells. Sperm were incubated for up to 180 min in capacitating conditions in the absence (C) or presence of Asta (2 µM), L1 (10 µg/mL), or both (Asta+L1). Luminol chemiluminescence was monitored during sperm capacitation. Results are expressed as moving averages of Relative Luminescence Units (RLU)/30 sec for 2 × 10^6^ cells. Figure is representative of *n* = 7 separate experiments. Detection was carried out in triplicate.

When the effects of the different treatments were assayed on the ROS generation curve (Figure 4), samples were incubated in the absence (C), or presence of Asta, or L1, or both (Asta+L1), and analyzed for ROS generation in a luminometer for 180 min with luminol as luminescent source. 

All samples showed similar curves of ROS generation compared to the control (C), thus suggesting that none of the treatments affected H_2_O_2_ formation in sperm. 

To initiate a successful infection, HPV must bind to heparan sulfate proteoglycan (HSPG) by exposing L1 from its capside structure and through L1 form an endocytic complex [25], that is also responsible for virus internalization [26]. 

It has been recently confirmed that also in human sperm, GAGs mediate the viral binding to the cell surface, in particular the GAG syndecan-1 (Synd-1) seems to interact with the viral capsid protein L1 of HPV16 [27,28]. Synd-1 belongs to the heparan sulfate GAGs (HSGAGs) family which regulates cell proliferation and cell-matrix and cell–cell adhesion by modulating the ligand-dependent activation of GAG receptors at the cell surface, interacting with components of the actin-based cytoskeleton with intracellular domain [29]. A direct interaction between the extracellular portion of Synd-1 and HPV-L1 is involved in L1 binding to sperm membrane, as confirmed by heparinase-mediated abolition of the co-localization of Synd-1 and HPV-1 [25,26].

In the present study, the L1 binding would act as an anchor complex, preventing further rafts translocation and relocation to the sperm head. This hypothesis is consistent with the results of Chen et al. [30] who demonstrated that Synd-1, clustered upon L1 ligand binding, induced recruitment and binding to cortactin, a protein involved in the membrane-cytoskeleton formation and modulation through actin–cortactin net interaction. From these considerations, we postulate that the probable binding site in human sperm membrane may be included in raft domains, thus being regulated and regulating, in turn, further L1 binding/inclusion and raft relocation. Asta, binding to plasma membranes, probably alters raft composition by sterically filling the loci dedicated to the interaction between external viral L1 and internal Synd.1. The possible mechanism underlying Asta-related induction of capacitation would rely on its ability to insert into the lipid bilayer, disengaging rafts from the blocks that maintain the membrane in non-capacitated form. Consistently, Asta would also release the protein links mediated by L1 and cytoskeleton proteins, both allowing/facilitating the correct engagement of proteins in rafts, such as in the case of actin polymerization [31], and the correct relocation of enzymes, such as Lyn, to the acrosome region. 

## 3. Experimental Section

### 3.1. Chemicals

Recombinant HPV16 L1 protein was purchased from Abcam (Cambridge, UK). Anti- HPV16 L1 mouse monoclonal, goat anti-mouse and anti-rabbit IgG- fluorochrome fluorescein isothiocyanate (FITC) conjugate antibodies were purchased from Santa Cruz Biotechnology (Heidelberg, Germany). Anti-P-Tyr mouse monoclonal and anti-Lyn rabbit polyclonal antibody were obtained by Upstate (Becton Dickinson Italia SpA, Milan, Italy) and Millipore (Temecula, CA, USA), respectively. Density gradient (Pure Sperm 40/80) and pure sperm wash buffer (PSW) were obtained from Nidacon International AB (Göteborg, Sweden). Asta was supplied by FERpharma s.r.l. (Milan, Italy). 12-myristate-13-acetate phorbol ester (PMA) was purchased from Calbiochem (Nottingham, UK) and all other reagents from Sigma-Aldrich (Milan, Italy).

### 3.2. Semen Collection and Analysis

Forty healthy male donors (age range: 25–43 years, average age: 34.6 years) were enrolled at the Centre of Assisted Reproduction-U.O.C. Obstetrics and Gynecology Clinic–Padua, Italy. After 3 days of abstinence, semen samples were collected by masturbation in a sterile container and then assessed for sperm parameters. All sperm samples used in this study were normal in terms of sperm count, motility, morphology, volume, and pH, according to the World Health Organization criteria [32]. All samples presenting any kind of contamination were discarded. This study was approved by the Ethics Committee for Research and Clinical Trials of our University (Code: 4400-AO18), and all recruited donors gave their informed written consent and provided detailed lifestyle histories.

### 3.3. Sample Preparation

After semen analysis, samples were laid on a discontinuous gradient (Pure Sperm 40%/80%) and centrifuged at 500× *g* for 30 min at room temperature. The seminal plasma and sperm from the 40% gradient interface were discarded, and the sperm cells from the bottom pellet (80% gradient) were gathered. After gradient separation, sperm samples were washed with PSW and collected three by three in a single pool (stock sample) to obtain a sufficient number of cells to perform all tests. Stock samples (concentration adjusted to 80 × 10^6^ sperm cells/mL in PSW) were divided in aliquots, analyzed immediately (T_0_), or incubated for up to 180 min in capacitating conditions, in the absence (C) or presence of Asta 2 µM (Asta) from stock solutions of 100 mM dissolved in dimethyl sulphoxide (DMSO), L1 1, 10 or 13 µg/mL (L1), or together with 30 min-Asta pre-incubation (Asta+L1 10).

### 3.4. Computer Assisted Sperm Analysis (CASA)

Sperm motility and hyperactivation were analyzed using a computer-assisted sperm analyzer (CASA, Ab scientific, London, UK). For each sample, the following parameters were evaluated: the percentage of motile spermatozoa and VAP (average path velocity), VSL (straight-line velocity), and ALH (amplitude of lateral head displacement) to determine the percentage of hyper-activated (HA) cells [33]. All measurements were performed at 37 °C. A minimum of 100 cells and 5 fields were analyzed for each aliquot.

### 3.5. Anti-L1, Anti-P-Tyr and Anti-Lyn Evaluations with Confocal Microscopy

Aliquots of sperm (15 × 10^6^ cells) from each sample were accurately washed with phosphate buffer saline (PBS) containing vanadate 1 mM and protease inhibitor cocktail, fixed with 2% (*w*/*v*) paraformaldehyde and incubated overnight at 4 °C on slides pre-coated with poly-L-lysine [20,21,22]. Slides were rinsed twice with PBS and sperm cells were permeabilized with 0.2% (*v*/*v*) Triton X-100 for 15 min at 4 °C and then incubated with anti-L1, anti-P-Tyr, or anti-Lyn antibodies for 1 h at 37 °C in a humid chamber. Slides were washed with PBS, stained with anti-mouse or anti-rabbit IgG-FITC conjugate for 1 h at 37 °C in a humid chamber and then rinsed with PBS and mounted. Staining without primary antibody was used as negative control. Fluorescence was detected with the UltraView LCI confocal system (Perkin Elmer, Waltham, MA, USA).

### 3.6. Evaluation of Membrane Rafts

GM1 membrane raft marker was visualized in live human spermatozoa by staining with the cholera toxin subunit B (CTB)-FITC [22,34]. For this purpose, suspensions of cells (15 × 10^6^ cells) from each sample were mixed with an equal volume of CTB (50 µg/mL) and incubated for 15 min at 37 °C. The sperm cells were then washed twice in PBS before being fixed in 2% paraformaldehyde for 30 min, mounted on poly-L-lysine coated glass microscope slides, and viewed using the confocal microscope as described above. For each treatment, at least 200 cells were counted. 

### 3.7. Evaluation of Acrosome Reaction

Acrosome status was monitored with acrosome-specific FITC-labeled peanut (*Arachis hypogaea*) agglutinin (FITC-PNA) in conjunction with DNA-specific fluorochrome propidium iodide (PI) as a viability test [23,24]. Briefly, in order to induce AR, aliquots (15 × 10^6^ cells) of each sample were incubated for 30 min at 37 °C, in the presence of 10 µM Ca^2+^ ionophore A23187. Samples containing DMSO, but not ionophore, were used as control. After incubation sperm cells were centrifuged, resuspended in PBS, and treated for 10 min at room temperature with 12 μM PI. Sperm was washed with PBS, fixed with 2% (*w*/*v*) paraformaldehyde and incubated overnight at 4 °C on poly-L-lysine-treated slides. Permeabilized sperm cells, as described above, were stained with 1 mg FITC-PNA/mL for 15 min at 37 °C in the dark, washed and mounted. At least 200 cells were evaluated for each sample, and fluorescence was detected as described above. Only sperm cells showing evenly distributed fluorescence over the acrosomal region were considered acrosome-intact.

### 3.8. Protein L1 Distribution

To determine whether and how much L1 was present in the plasma membrane, head or flagellum, intact spermatozoa (30 × 10^6^ cells) from each treatment were accurately washed in PBS-VI (containing vanadate 1 mM and protease inhibitor cocktail), resuspended in 200 μL of the same PBS-VI, and sonicated 3 times (30 s followed by a 10-s rest period each) on ice. Heads and flagellar fragments were then separated by a 15-min centrifugation (700× *g*) at 4 °C through a 75% Percoll layer in PSW. Flagellar fragments were recovered at the surface of the Percoll layer while the heads were found in the pellet. The purity of each fraction was assessed by microscopy prior to proceeding to analysis. The supernatant was centrifuged for 10 min (10,000× *g*, 4 °C) and the resulting supernatant was further centrifuged (100,000× *g*) to separate the membrane from the cytosol [21]. Each resulting fraction (membranes M, cytosol C, head H and flagella F) was diluted with PBS to the initial volume except for membranes, which were resuspended in 30 μL of PBS-VI. The presence of L1 protein was investigated by Western blotting and immuno-revealed with anti-L1 antibody.

### 3.9. Western Blotting

Cell fractions (membrane, heads and tails) or corresponding cytosol (Figure S1) were solubilized by adding SDS and β-mercaptoethanol (2% final concentration), boiled at 100 °C for 5 min, and subjected to SDS/PAGE (10% polyacrylamide gels). Proteins were electrotransferred to a nitrocellulose membrane and immunorevealed with anti-L1 antibodies. Loading control was performed with anti-actin antibodies [35].

Densitometric analysis. Bands corresponding to L1 protein bands were counted by ImageJ software 1.48v.

### 3.10. ROS Enhanced Chemiluminescence (ECL)

Production of ROS was measured by the chemiluminescence assay method with luminol (5-amino-2,3-dihydro-1,4-phthalazinedione) as probe [23,24]. Briefly, 2 µL of 25 mM luminol and 4 µL of 10 mg/mL horseradish peroxidase, both prepared in DMSO, were added to 200 μL of a sperm suspension at a concentration of 10 × 10^6^ cells/mL. ROS levels were determined by a luminometer (Fluoroskan Ascent FL, Labsystems, Helsinki, Finland) in the integrated mode for 180 min at 37 °C. Results are expressed as Relative Luminescence Units (RLU) per 2 × 10^6^ sperm cells. Lastly, 2 µL of a 10 mM N-formylmethionyl-leucyl-phenylalanine (FMLP) stock was added and, after a further 10 min of incubation, 4 µL of a 1 nM stock solution of PMA was added, to exclude leukocyte contamination. Only samples with negative response to FLMP and PMA were processed.

### 3.11. Statistical Analysis

Results are expressed as means ± SD. Comparisons among multiple groups were obtained with ANOVA followed by Dunnett’s test. Statistical significance was set at *p* < 0.05 (two-tailed). Statistical analysis were performed with JMP^®^ 13 software (SAS Institute, Cary, NC, USA).

## 4. Conclusions

Recent evidence substantially strengthens the possibility that HPV is responsible not only for HPV-associated cancers, but also for idiopathic infertility due to its detrimental effect on sperm parameters [36,37,38] and the potential stage arrest of embryo development [39]. In this study we showed that L1, at 10 μg/mL, binds to quit 100% of sperm membrane, locating at the sides of mid-piece and head. This protein, blocks rafts translocation, Lyn displacement, and Tyr-P of the head, thus resulting in a dramatic decrease of the percentage of ARC. Asta treatment prevented L1 binding to the membranes reducing by more than 50% the presence of L1, thus protecting cells from HPV L1 binding. Further studies are needed to better clarify the mechanism of HPV L1 entry, and Asta could be considered for its potential antiviral effect.

## Figures and Tables

**Table 1:** Sperm motility and kinematic parameters observed in different samples. Motility and kinematic parameters of spermatozoon were evaluated with computer-assisted sperm analysis (CASA) at T_0_ (before starting incubation) and after 180 min of incubation in capacitating conditions in absence (C) or presence of L1, Asta or both. Motility = progressive and non-progressive motility (%); VSL = straight-line velocity (µm/s); VAP = average path velocity (µm/s); ALH = amplitude of lateral head displacement (µm).

	Motility (%)	VSL (µm/s)	VAP (µm/s)	ALH (µm)
T_0_	68 ± 9	58.4 ± 8.9	54.0 ± 6.7	3.1 ± 0.5
C	75 ± 11	77.8 ± 13.9 ^a^	67.6 ± 11.0 ^a^	4.9 ± 0.9 ^a^
L1 1 µg/mL	74 ± 10	76.9 ± 9.4 ^a^	67.3 ± 9.0 ^a^	4.7 ± 0.6 ^a^
L1 10 µg/mL	73 ± 8	76.3 ± 8.8 ^a^	67.5 ± 7.2 ^a^	4.8 ± 0.7 ^a^
L1 13 µg/mL	73 ± 8	76.4 ± 8.9 ^a^	67.4 ± 8.1 ^a^	4.8 ± 0.6 ^a^
Asta	75 ± 9	78.2 ± 13.6 ^a^	67.9 ± 9.3 ^a^	5.0 ± 0.6 ^a^
Asta+L1 10	74 ± 6	77.7 ± 11.3 ^a^	67.0 ± 7.5 ^a^	4.9 ± 0.7 ^a^

^a^: *p* < 0.001, comparing each parameter under different treatment against T_0_, by using Dunnett’s test, following a significant one-way ANOVA; no significant difference was observed comparing each treatment against C. Values are expressed as the mean ± SD.

**Table 2:** Sperm biochemical parameters observed in different samples. Sperm biochemical parameters were evaluated with fluorescence microscopy at T_0_ (before starting incubation) and after 180 min of incubation in capacitating conditions in absence (C) or presence of L1 (L1 1, 10 or 13 µg/mL), Asta or both (Asta+L1 10 µg/mL) as described in Methods.

	L1 (%)		CTB (%)	Lyn (%)	Tyr-P (%)	ARC (%)	NVC (%)
	Tot. Stained	Head	Head	Head	Head		
T_0_	ND	ND	13.4 ± 1.7	9.2 ± 1.2	15.3 ± 2.1	8.1 ± 1.3	7.1 ± 1.2
C	ND	ND	72.0 ± 3.6	63.1 ± 2.5	68.4 ± 2.9	65.7 ± 4.2	10.1 ± 0.9
L1 1 µg/mL	55.3 ± 3.6 ^a^	17.6 ± 7.0 ^a^	25.5 ± 3.5 ^a^	20.5 ± 2.7 ^a^	19.7 ± 2.3 ^a^	20.9 ± 3.2 ^a^	10.3 ± 1.1
L1 10 µg/mL	98.1 ± 1.6 ^a^	59.9 ± 3.9 ^a^	21.3 ± 4.2 ^a^	16.3 ± 3.5 ^a^	11.3 ± 1.7 ^a^	19.4 ± 2.8 ^a^	10.6 ± 1.3
L1 13 µg/mL	99.1 ± 0.7 ^a^	62.1 ± 4.8 ^a^	15.8 ± 3.5 ^a^	13.9 ± 2.2 ^a^	10.2 ± 1.3 ^a^	14.6 ± 3.3 ^a^	11.4 ± 1.0 ^b^
Asta	ND	ND	72.9 ± 2.8	65.4 ± 2.8	71.3 ± 1.8 ^b^	67.9 ± 3.5	9.3 ± 1.5
Asta+L1 10	45.5 ± 4.2 ^a,c^	43.8 ± 9.0 ^a,c^	50.2 ± 2.7 ^a,c^	43.2 ± 3.0 ^a,c^	43.6 ± 3.9 ^a,c^	40.8 ± 2.6 ^a,c^	9.6 ± 1.0

^a^: *p* < 0.001, ^b^: *p* < 0.05, comparing each sample against C by using Dunnett’s test, following a significant one-way ANOVA; ^c^: *p* < 0.001, comparing Asta+L1 10 sample to L1 10 µg/mL by using Dunnett’s test, following a significant one-way ANOVA. ND: not detectable, and assumed as zero for calculation. Values are expressed as the mean ± SD.

## Supplementary Materials

The following are available online at http://www.mdpi.com/1660-3397/16/11/427/s1, Figure S1: Detection of protein L1 in different fractions of human sperm capacitated in presence of L1, Figure S2: Detection of protein L1 in cytosol of human sperm during capacitation in absence or presence of L1 and/or Asta.
