# Supplementary material for: Astaxanthin Prevents Human Papillomavirus L1 Protein Binding in Human Sperm Membranes
Source: Mar Drugs. 2018 Nov 2;16(11):427. doi: 10.3390/md16110427 (PMC6266165; doi:10.3390/md16110427)
Supplement: Supplementary file 1 [file marinedrugs-16-00427-s001.docx]

**Figure 1 Supplementary**


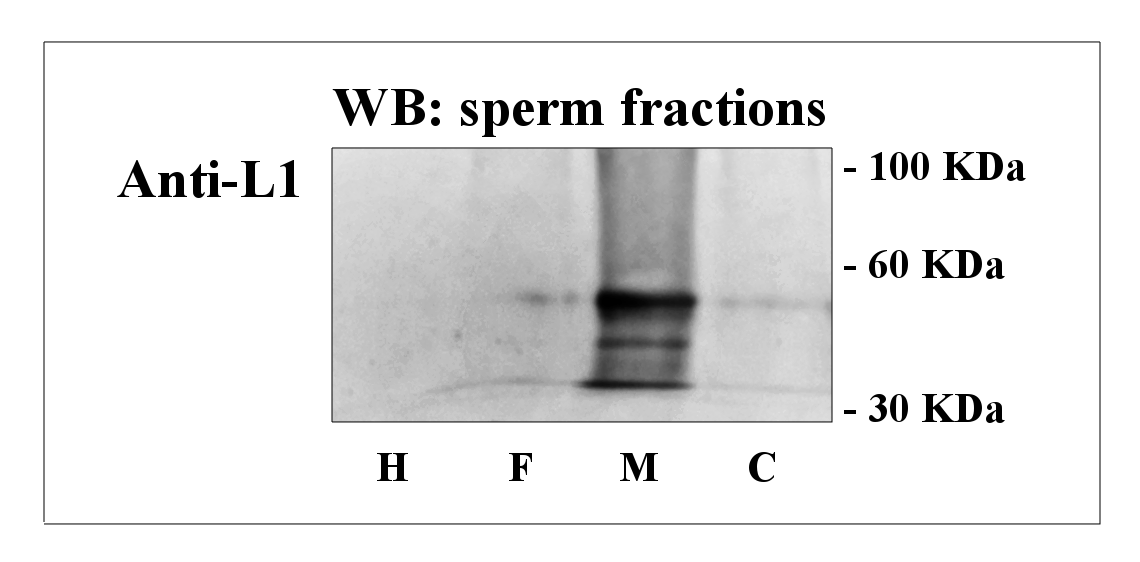


**Figure 1 Supplementary.** **Detection of protein L1 in different fractions of human sperm capacitated in presence of L1.** Western blot analysis of cells fractions (head H, flagella F, membranes M and cytosol C, obtained as described in Methods) of sperm, incubated in capacitating conditions for 180 min in presence of L1 10 μg/μl. Intact spermatozoa (30×10^6^ cells) were accurately washed and sonicated 3 times on ice. Heads and flagellar fragments were then separated by a 75% Percoll gradient. Flagellar fragments were recovered at the surface of the Percoll layer and the heads were found in the pellet. The supernatant was further centrifuged to separate the membrane from the cytosol. Different fractions were analyzed by SDS-PAGE, transferred to nitrocellulose and immuno-revealed with anti-L1 antibody. The figure is representative of seven separate experiments conducted in triplicate.

**Figure 2 Supplementary**


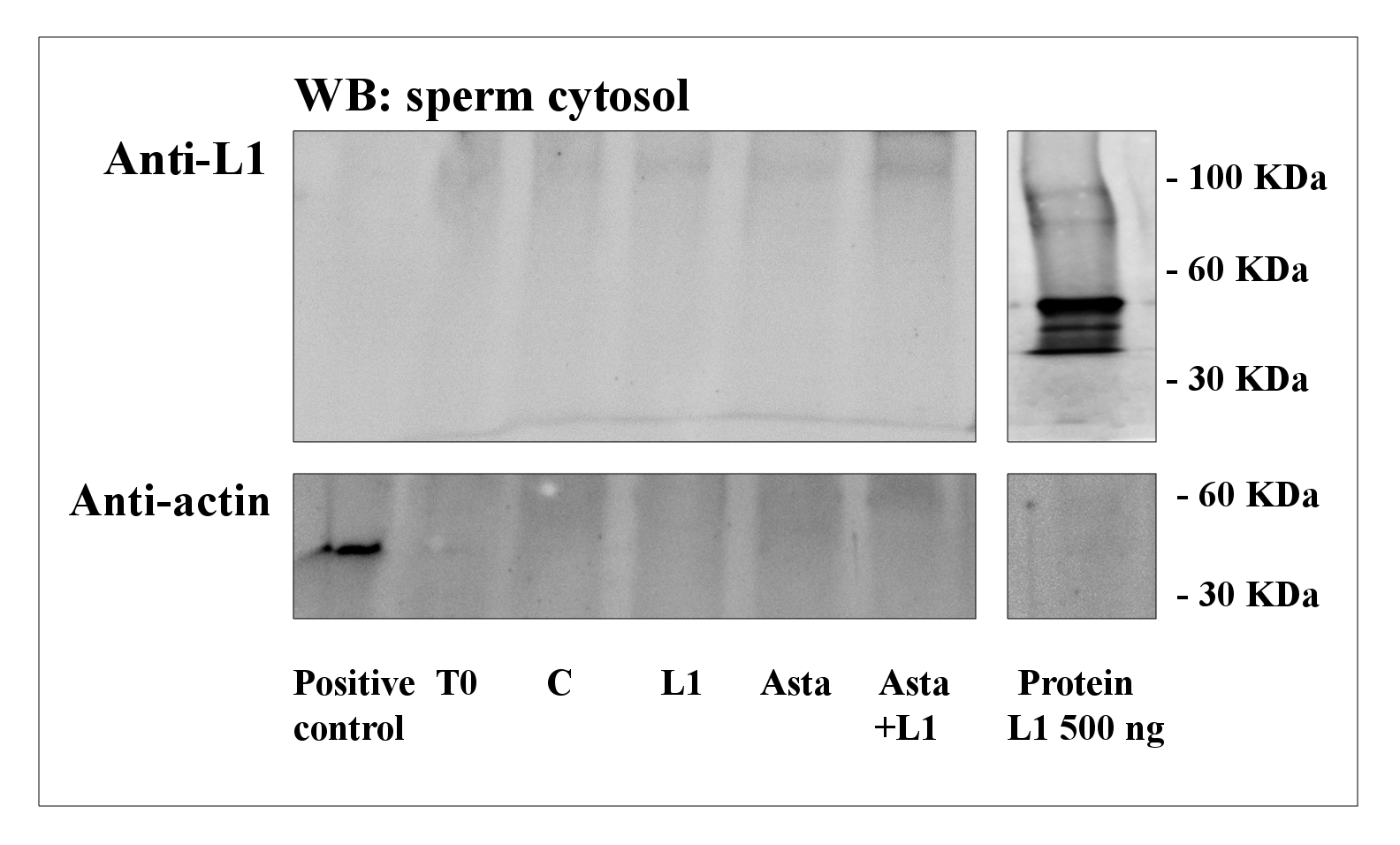


**Figure 2 Supplementary.** **Detection of protein L1 in cytosol of human sperm during capacitation in absence or presence of L1 and/or Asta.** Western blot analysis of cytosol (obtained as described in Methods) of sperm cells, at T_0_ or incubated in capacitating conditions for 180 min in absence (C) or presence of L1 10 μg/μl, Asta 2 μM or Asta+ L1. Cytosol of different samples were analyzed by SDS-PAGE, transferred to nitrocellulose and immuno-revealed with anti-L1 antibody (L1 protein 500ng) and then with anti-β actin (Positive control: Red blood cells). The figure is representative of seven separate experiments conducted in triplicate.
